# Mapping quantitative trait loci (QTL) in sheep. II. Meta-assembly and identification of novel QTL for milk production traits in sheep

**DOI:** 10.1186/1297-9686-41-45

**Published:** 2009-10-22

**Authors:** Herman W Raadsma, Elisabeth Jonas, David McGill, Matthew Hobbs, Mary K Lam, Peter C Thomson

**Affiliations:** 1 ReproGen - Animal Bioscience Group, Faculty of Veterinary Science, University of Sydney, 425 Werombi Road, Camden NSW 2570, Australia

## Abstract

An (Awassi × Merino) × Merino backcross family of 172 ewes was used to map quantitative trait loci (QTL) for different milk production traits on a framework map of 200 loci across all autosomes. From five previously proposed mathematical models describing lactation curves, the Wood model was considered the most appropriate due to its simplicity and its ability to determine ovine lactation curve characteristics. Derived milk traits for milk, fat, protein and lactose yield, as well as percentage composition and somatic cell score were used for single and two-QTL approaches using maximum likelihood estimation and regression analysis. A total of 15 significant (*P *< 0.01) and additional 25 suggestive (*P *< 0.05) QTL were detected across both single QTL methods and all traits. In preparation of a meta-analysis, all QTL results were compared with a meta-assembly of QTL for milk production traits in dairy ewes from various public domain sources and can be found on the ReproGen ovine gbrowser http://crcidp.vetsci.usyd.edu.au/cgi-bin/gbrowse/oaries_genome/. Many of the QTL for milk production traits have been reported on chromosomes 1, 3, 6, 16 and 20. Those on chromosomes 3 and 20 are in strong agreement with the results reported here. In addition, novel QTL were found on chromosomes 7, 8, 9, 14, 22 and 24. In a cross-species comparison, we extended the meta-assembly by comparing QTL regions of sheep and cattle, which provided strong evidence for synteny conservation of QTL regions for milk, fat, protein and somatic cell score data between cattle and sheep.

## Background

Sheep represent an economically important agricultural resource in the global meat, fibre, and milk production systems of both the developed and developing world. The multi-purpose nature of many sheep breeds and the highly specialised single purpose breeds, demonstrate the versatility and suitability of sheep production in a diverse set of production systems [1]. Sheep milk production represents a specialised commodity which has been developed across many breeding systems in either dual purpose, synthetic composite lines, or specialised dairy breeds such as the Awassi, Chios, Comisana, Lacaune, Laxta and Sarda Breeds [2]. Genetic variation has been reported for most of the major milk traits, and has been successfully exploited in genetic improvement programmes for sheep dairy production [2-6].

Over the past few decades, numerous quantitative trait loci (QTL) studies have been conducted on many breeds of livestock to enrich our knowledge on the underlying biology and genetic architecture of complex traits. A general review of QTL mapping can be found in Weller [7]. However for milk production and udder health, fewer QTL studies have been conducted [3,8-12] in dairy sheep in comparison with cattle, as reviewed by Khatkar et al. [13] and summarised on the animal genome website [14]. Analysis of QTL information from diverse sources within a species provides important information on the consistency and utility of QTL across breeds, and is amenable to meta-analysis to reach a consensus on QTL location and effect [13]. Furthermore, cross-species comparative analyses with a species for which many QTL for the same traits have been reported such as milk traits in cattle may provide additional insight to QTL in a closely related species. This may give insight into ancestral genes for economically important traits and adds information to accelerate fine-mapping in the QTL information-sparse species.

QTL mapping requires robust phenotypes. In the case of lactation measurements, the nature of these data is heterogeneous with respect to frequency, length and regularity of recording. One approach is to use model-based prediction to standardise lactation characteristics. The advantages are that the heterogeneity mentioned above as well as age, parity and other effects are taken into account by producing a standardised curve, further facilitating meaningful comparisons. Using model-based predictions rather than empirical data has also the advantage of minimising random variation while simultaneously summarising the lactation profile into biologically interpretable parameters [15]. Therefore these derived parameters are then useful for linkage studies since they describe lactation-wide or lifetime milk production. One of the first lactation models used in dairy cattle was developed by Brody et al. [16] and is based on an exponential decay function. Further models were also proposed by Sikka [17], Wood [18], Cobby and Le Du [19], and Cappio-Borlino et al. [20]. By contrast, modelling lactation curves in dairy sheep is far less common [21-25], but it is expected that dairy cattle models can be applied to sheep lactations [26].

This study reports an appropriate model to describe the characteristics of the sheep lactation curve, provides additional information on QTL for milk traits in sheep, and provides a within and cross-species QTL analysis from publicly available information in sheep and cattle.

## Methods

### Resource population

As described by Raadsma et al. [27] a resource population from crosses between the improved dairy type Awassi (A) and the apparel wool Merino (M) sheep was established to exploit the extreme differences between these two sheep types in a range of production characteristics. The improved Awassi sheep was developed in Israel and has been identified as an ideal breed for milk production [28] and possesses fleece characteristics suitable for carpet wool, whilst the Merino sheep was originally developed for the production of high quality apparel wool and recently used for meat production [1] but is a poor milk producer. The Merino breed [29,30] with its low milk yield, and the Awassi breed with its medullated carpet-wool quality represent two extremes for these production traits. Further details on the development of the resource population can be found in Raadsma et al. [27]. For modelling the lactation curves, lactation data from different generations, AMM backcross (AMM), AM_AMM double backcross (DBC) and intercross (INT) progeny (*n *= 622), were used. In the QTL study reported here, only genotypic information from the 172 ewe G_2 _AMM progeny of the first F_1 _sire were available where a genome-wide scan was performed. The additional families will be used in future work to confirm QTL effects and to fine-map confirmed QTL in combination with high density SNP marker analysis.

### Marker analysis

A genome-scan using 200 polymorphic microsatellite markers covering all 26 autosomes was conducted in 172 backcross ewes. The markers comprised 112 cattle (*Bos taurus*) markers, 73 sheep (*Ovis aries*) markers, and 15 other Bovinae markers. The procedures for DNA extraction, genotyping and allele calling, are described in detail in the first paper of this series [27]. All markers used for this study were the same as those used in the first paper of the series, and were mapped to the previously described population specific framework map [27].

### Milk recording

Milk yields were recorded from morning and afternoon milking from 1999 to 2007 (except from 2004 to 2006 when only one daily milking was conducted). These yields were recorded using a Tru-Flow meter (Tru Flow Industrial Pumps, Bathurst NSW Australia) in the first phase until 2001 and subsequently with SRC-Tru Test electronic meter (Tru-Test Pty Limited, Mentone VIC Australia). Recordings were made daily in the first phase of the study and subsequently three times per week in the latter phase of the experiment. Milk from morning and afternoon milking was sampled for composition analyses to determine protein (PP), fat (FP), and lactose (LP) percentage and somatic cell count (SCC) in cells/mL. The somatic cell score (SCS) was calculated as the natural logarithm of SCC. Samples were analysed by 'Dairy Express' Australia [31].

### Analysis of lactation data

Amongst a selection of lactation curve models that have been proposed in the literature [16-20], the Wood [18] model was selected here because of its simplicity and flexibility to derive key parameters that can be used to describe specific characteristics of the lactation curve. From a cursory check it was not obvious that other models provided a better fit to the data. The Wood model is defined as follows:

where *W*(*t*) is the expected milk yield at time *t *expressed in litres/day; *t *represents the time in days after parturition; *a *is a scaling parameter related to total yield of lactation with *k *= ln(*a*); parameter *b *is related to the rate of increase prior to the lactation peak; and *c *is a parameter related to the rate of increase after the lactation peak. From this model, the cumulative milk yield up to day *T *(e.g. 100 days) can be calculated numerically as

where γ(·) is the lower incomplete gamma function, , and can be evaluated by its relation to the cumulative distribution function of a gamma distribution.

To fit lactation curve models to multiple sheep simultaneously, the Wood model was fitted using a nonlinear mixed model by the nlme() function in R using the methods documented in Pinheiro et al [32]. In this case the model fitted is:

where *y*_*it *_is the milk yield at time *t *for sheep *i*, *k*_*i *_*b*_*i *_and *c*_*i *_are the Wood model "parameters" for sheep *i*; and ε_*it *_*is *the random error of sheep *i *at time *t*. These sheep-specific "parameters" can each be written as *k*_*i *_= κ_*i *_+ *K*_*i*_, *b*_*i *_= β_*i *_+ *B*_*i *_and *c*_*i *_= χ_*i *_+ *C*, where κ_*i*_, *β *_*i *_and χ_*i *_are the fixed effects and *K*_*i*_, *B*_*i *_and *C*_*i *_are random deviations from these fixed effect means, assumed to have a multivariate normal distribution,

The non-zero covariance terms (σ_*KB*_, σ_*KC*_, σ_*BC*_) have been included to allow for correlations amongst the "parameters", as observed in initial exploratory analyses. Note that the choice of the parameterisation of the Wood model as (*k*, *b*, *c*) rather than as (*a*, *b*, *c*) was made based on the closer approximation of the resultant deviations to a multivariate normal distribution. Also, the choice to analyse the yield (*y*_*it*_) as a nonlinear mixed model, rather than ln(*y*_*it*_) as a linear mixed model was made on the basis of residual diagnostics. The output from this analysis was to produce model-based 100-day cumulative milk yields for each ewe-lactation, adjusted to a common set of fixed effects. Preliminary studies using information of these animals have shown that the 100-day lactation performance is highly correlated with the extended lactation (200 to 400 days), whereas 50 and 80 day lactation performances were unreliable in predicting extended lactation performance (additional file 1). Therefore, due to the differences in recording durations across individual ewe-lactations, we chose to use model-based 100 day cumulative yields to describe and standardise the lactation performance of all ewes.

Values for PP, FP, LP, SCC and SCS were analysed by fitting a linear mixed model using the lme() function in R. As suggested by Barillet [2], the useful yield describes the ability to process milk into cheese, and was calculated using the predicted protein and fat percentages from the mixed model where UP = FP + 1.85 PP [2]. The cumulative milk yield at day 100 (YCUM) for each animal was multiplied with the milk content (protein, fat, lactose) and somatic cell scores/counts, to calculate the cumulative milk content until day 100 (PYCUM, FYCUM, LYCUM, YSCC, and YSCS for protein, fat, lactose, somatic cell scores and somatic cell counts, respectively).

### QTL mapping procedure

QTL analyses were performed for all traits using two methods. Solutions were first obtained using the QTL-MLE procedure in R as described previously [27,33]. To account for multiple testing and to minimise the number of false positive QTL, the method developed by Benjamini and Hochberg [34] was used to calculate genome-wise ranked *P*-values for each trait. For QTL-MLE, a LOD 1.75-2.0 was deemed suggestive, LOD 2-3 significant, and LOD greater than 3 highly significant. The second method used the regression analysis for half-sib design in the web-based program QTL Express [35]. For this method, QTL with chromosome-wide significance threshold (*P *< 0.05) were described as suggestive, chromosome-wide levels *P *< 0.01 as significant and experiment-wide levels (*P *< 0.05 and *P *< 0.01) as highly significant QTL. Thresholds for QTL-MLE and QTL Express were chosen according to the threshold criteria applied in the first paper of this series. A two-QTL model was also fitted to the data using the same program [35]. The QTL heritability was calculated as the proportion of the phenotypic variance accounted for by the QTL [1-(mean square of full model/mean square of reduced model)].

### Power analysis

Based on a Type I error of 0.05, the design had a predicted power of 0.72 to detect QTL with 0.4 SD effect [36]. In addition, the observed power for QTL detected under the Haley-Knott regression method was calculated using the method described by Hu and Xu [37]. The power was calculated at two different significance thresholds namely, *P *< 0.05 and *P *< 0.01.

### Meta-assembly

A meta-assembly of QTL identified in this study was conducted by collating all known QTL from public sources for matched traits. Due to fewer records in sheep, it was not possible to conduct a meta-analysis as described for cattle by Khatkar et al. [13] to obtain consensus on the number and positions of QTL by means of a formal statistical hypothesis-based testing procedure. In contrast, for a qualitative assessment, a meta-assembly was undertaken by standardising all QTL against the V4.7 sheep linkage map [38,39]. For each QTL, we identified the markers closest to the likely point location and to the ends of the 95% confidence interval (CI) segment. When no point location is available we used the midpoint of the chromosome. These markers or co-located markers were found on the reference map. For each QTL we defined a weighting function which gives a score which is maximal (1.0) at the point location and follows a quadratic decline to 0.1 at the boundaries of the CI segment. For each trait we summed the scores of non-redundant QTL. QTL identified as being potential duplicates of the same QTL (*i.e*. QTL identified in the same study within an identical marker interval by different methods) were defined as redundant reported QTL. The individual QTL locations and their scores, and meta-score profiles were loaded into the ReproGen gbrowse GFF database [40] which can be browsed at http://crcidp.vetsci.usyd.edu.au/cgi-bin/gbrowse/oaries_genome/. This browser includes hyperlinks to the detailed QTL information for each locus. In a similar way we constructed a bovine QTL meta-assembly using previously published estimates of cattle QTL as reviewed by Khatkar et al [13]. Bovine QTL were extracted from the ReproGen QTL database described by Khathar et al. [13]. For a comparative analysis of ovine and bovine QTL we first defined blocks of synteny by comparing the locations of markers in the ovine reference map with their positions in the bovine btau4.0 genome sequence assembly [41]. The syntenic blocks can be used to compare features across genomes. By this means QTL from our bovine meta-assembly were added to the ReproGen ovine QTL browser. Finally we combined the ovine and bovine meta-scores to give a two-species meta-score and this also was made available as a track in gbrowse.

## Results

### Analysis of lactation data

**Figure 1:**
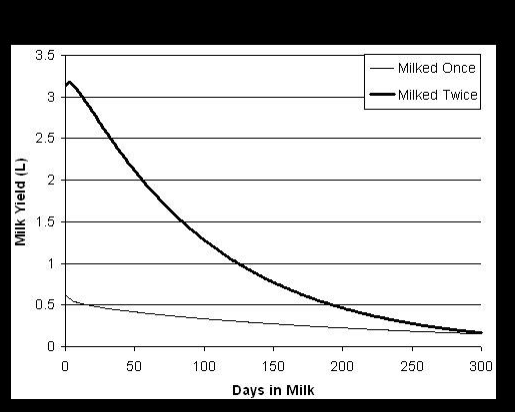
**Example milking curves for ewes milked once compared to twice daily**. Shown are the total milk yield in litres (L) at the lactation day (days in milk) for animals milked twice or once a day

In total 1509 lactation records with more than 130,000 observations from 622 ewes were obtained across the experiment and the Wood lactation model was then fitted to these data. A range of one to five lactations was available for each ewe. In the present study, the ewes were either milked once or twice a day over the lactation, leading to a considerable difference of the shape of the lactation curves and total milk yields (Figure 1). The total milk yields were standardised against birth type = 1 (singleton), third parity, twice milking/day. The fixed effect of season was significant in all models. Days in milk, sire, genetic group, and parity were non significant effects for all traits analysed. Age of animal, birth type, year of milking, milk yield and milking frequency were significant for specific traits. The number of observations, mean, standard deviation and the range values of all traits recorded for the 172 genotyped backcross females on which the QTL analysis were performed, are presented in Table 1. The YCUM of the lactation ranged between 26.1 and 126.3 kg with an average of 73.3 kg. The PYCUM ranged between 1.46 and 6.81 kg (average 4.05 kg). For the FYCUM a mean of 4.86 kg was found with yields ranging between 1.7 and 8.12 kg and for LYCUM ranged between 0.93 and 4.49 kg (average 2.62 kg). The mean PP and FP was 5.54%, and 7.09% respectively, and mean LP was 3.56% with almost no variance for the latter. The SCS ranged between 3.87 and 7.40 (average 4.97), and the SCC had an average of 396 cells. The useful yield content was between 15.3 and 19.25 (average 17.33).

**Table 1:** Summary of the phenotypic lactation traits in 172 ewes used for QTL mapping

	N	Mean	sd	Min	Max
Fat content [%]	156	7.1	0.57	5.3	8.2
Lactose content [%]	156	3.6	0.00	3.6	3.6
Protein content [%]	146	5.5	0.20	4.9	6.1
YCUM [kg]	156	73	19	26	126
PYCUM [kg]	139	4.2	1.1	1.8	6.9
FYCUM [kg]	145	5.2	1.6	1.6	9.0
LYCUM [kg]	145	2.6	0.70	0.93	4.5
YSCS	148	3.6	1.0	1.3	6.5
YSCC	145	287	216	71	1607
SCS	159	5.0	0.46	3.9	7.4
SCC [cells/mL]	156	396	294	192	2408
Useful yield [%]	146	17	0.79	15.3	19

Shown are the number of observations (N), the average (Mean), standard deviation (sd), minimum (Min) and maximum (Max) value of the total milk (YCUM), protein (PYCUM), fat (FYCUM), lactose (LYCUM) yield until day 100, total somatic cell count (YSCC) and total cell score (YSCS) until day 100

### Putative QTL identified for lactation performance

**Figure 2:**
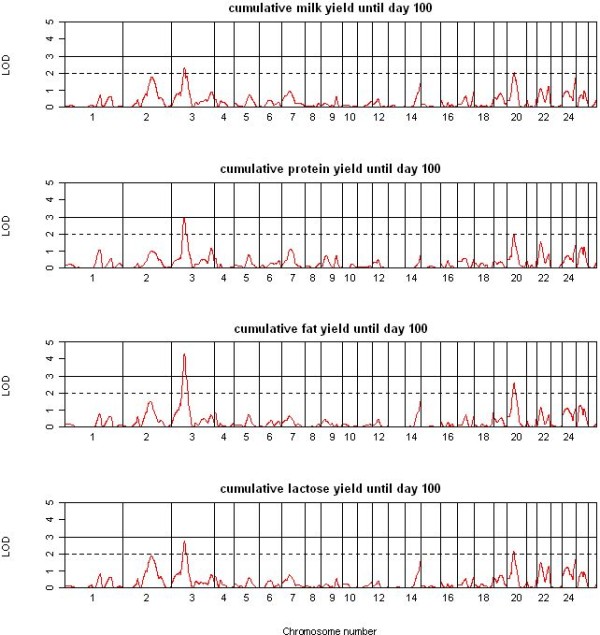
**Results of the QTL analysis using QTL-MLE for the single traits of the yields until day 100**. Drawn are the genome-wide graphs for the single traits of the cumulative yields until day 100 for milk, protein, fat and lactose; chromosomes are separated by vertical lines and further the significance thresholds (LOD = 2 and LOD = 3) are shown in each graph

**Figure 3:**
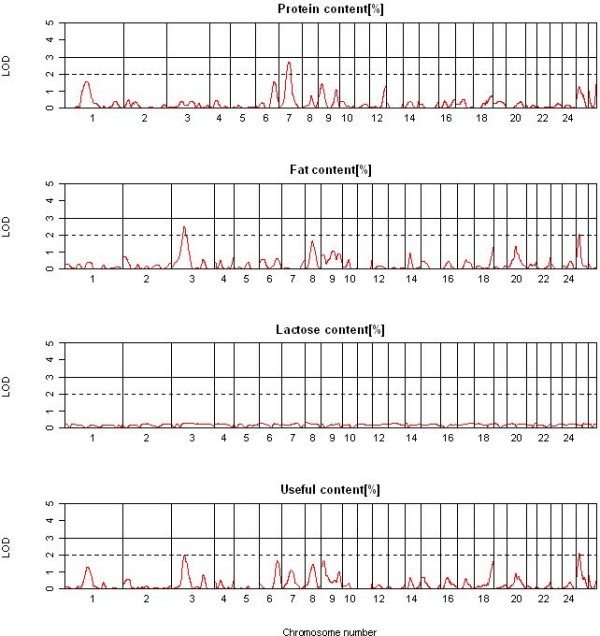
**Results of the QTL analysis using QTL-MLE for the single milk content traits**. Drawn are the genome-wide graphs for the single milk contents traits for protein, fat and lactose and the useful yield; chromosomes are separated by vertical lines and further the significance thresholds (LOD = 2 and LOD = 3) are shown in each graph

**Figure 4:**
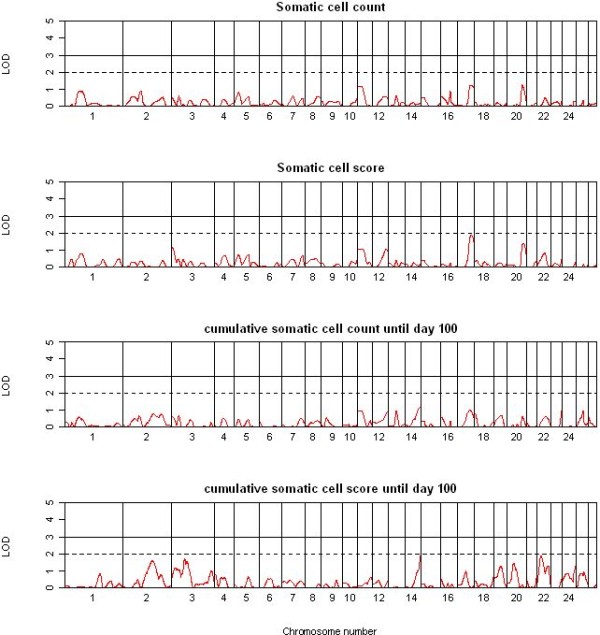
**Results of the QTL analysis using QTL-MLE for the single traits of the somatic cell count and score**. Drawn are the genome-wide graphs for the single traits of the somatic cell count and score and the cumulative somatic cell count and score; chromosomes are separated by vertical lines and further the significance thresholds (LOD = 2 and LOD = 3) are shown in each graph

A total of 40 suggestive and significant QTL were detected across the two single QTL analyses methods and 12 traits. All 13 significant and 11 suggestive QTL detected by QTL-MLE (Table 2) were also identified using QTL Express (additional file 2). The genome locations of the significant QTL for the four yield traits (YCUM, PYCUM, FYCUM, and LYCUM) are shown in Figure 2, with OAR3 and OAR20 showing a significant QTL for all four traits. OAR2 showed suggestive QTL for YCUM and LYCUM. The genome-wide QTL plots for the four composition traits (PP, FP, LP and UP) are shown in Figure 3. OAR3 and OAR25 showed significant QTL for FP and UP whereas OAR7 harboured a significant QTL for PP. A remarkably flat QTL profile was observed for LP with no significant QTL detected across the genome. No significant and relatively few suggestive QTL were detected for SCC, SCS, YSCS and YSCC (Figure 4) with the strongest support for QTL for SCS on OAR17 and for YSCS on OAR14 and 22. The detailed information of the locations, effect sizes and confidence intervals for the 24 QTL is shown in Table 2 for QTL-MLE. Allelic effects for the significant QTL ranged from -0.96 SD to 0.77 SD for QTL recorded by QTL-MLE.

**Table 2:** Summary of QTL for lactation traits using QTL-MLE

OAR	Trait	QTL position [cM]		CI [cM]		LOD	SD
				
		Peak^1^	Marker	Lower^1^	Upper^1^		
2	LYCUM	211	CSRD254	162	252	1.9	0.77
2	YCUM	214	CSRD254	182	253	1.8	0.73

3	FP	96	DIK4796	76	121	2.5	-0.67
3	FYCUM	96	DIK4796	88	104	4.4	-0.96
3	LYCUM	96	DIK4796	82	116	2.7	-0.74
3	U	96	DIK4796	77	124	2.0	-0.59
3	YCUM	96	DIK4796	80	123	2.3	-0.65
3	PYCUM	94	DIK4796	82	105	3.0	-0.79

6	UP	134	DIK4796	112	155	1.7	-0.53

7	PP	76	MCM223	56	93	2.7	0.74

8	FP	57	UWCA9	30	84	1.6	-0.49

9	UP	18	ETH225	7.2	49	1.7	-0.63

14	YSCS	114	MCMA19	91	114	2.0	0.57
14	LYCUM	114	MCMA19	82	114	1.6	0.51

17	SCS	102	BM7136	82	127	1.9	-0.55

20	FYCUM	50	DYAB	35	69	2.6	0.61
20	LYCUM	49	DYAB	33	68	2.1	0.55
20	PYCUM	49	DYAB	33	70	1.9	0.53
20	YCUM	52	DYAB	32	74	2.0	0.54

22	YSCS	23	BMS907	10	55	1.9	0.55

24	YCUM	105	DIK5147	84	105	1.9	0.51
24	LYCUM	105	DIK5147	82	105	1.8	0.51

25	FP	21	DIK2451	6	31	2.0	-0.51
25	UP	21	DIK2451	7	35	2.1	-0.54

QTL detected for the milk traits in the AMM BC population for the following traits: protein (PP), fat (FP), lactose (LP) and useful yield (UP) content, total milk (YCUM), protein (PYCUM), fat (FYCUM), and lactose (LYCUM) yield until day 100, total somatic cell count (YSCC) and total cell score (YSCS) until day 100; shown are the QTL relative QTL position and the confidence interval (CI) along the ^1^male distance map [27], the LOD score and the effect in standard deviation (SD) of the QTL; QTL effects are expressed with respect to the ancestry of Awassi grandsire contrasted with the Merino granddam

Using the regression analysis two additional significant QTL (exceeding the 1% chromosome-wide significance level) were identified, both of them did not reach the significance threshold using QTL-MLE. An additional 16 suggestive QTL were identified on OAR1, 2, 5, 6, 11, 13, 14, 17, 20, 22, 23, 24 and 26 (additional file 2) for all 12 traits using QTL Express. The highest phenotypic variance explained by QTL using QTL Express was for FYCUM on OAR3 (10.7%). The other significant QTL explained between 5.6 and 8% of the phenotypic variance (additional file 2).

QTL detected under either method, showed large confidence intervals as determined by 1-LOD support intervals under QTL-MLE (Table 2) or from bootstrap procedures under QTL Express (additional file 2). Among the results, QTL within the same marker interval were identified for the yield traits including milk, protein, fat, lactose, and somatic cell score yield. The derived traits of protein yield, fat yield, lactose yield, somatic cell count and somatic cell score yield are strongly correlated with milk yield. Phenotypic correlations in the range of 0.94 to 1 were detected between the yield traits (Table 3). This suggests that a general QTL for milk yield may affect the QTL for protein, fat, lactose yield and for total somatic cell score.

**Table 3:** Phenotypic correlation between the lactation traits used for the QTL analysis

	SCS	FP	PP	SCC	FYCUM	LYCUM	LP	PYCUM	UP	YCUM	YSCS
FP	-0.22										
PP	-0.21	0.42									
SCC	0.88	-0.19	-0.12								
FYCUM	-0.09	0.53	0.33	-0.05							
LYCUM	-0.04	0.29	0.24	0.01	0.96						
LP	-0.07	0.00	-0.03	-0.04	0.02	0.02					
PYCUM	-0.06	0.33	0.37	-0.01	0.97	0.99	0.02				
UP	-0.26	0.91	0.76	-0.19	0.53	0.32	-0.01	0.41			
YCUM	-0.04	0.29	0.24	0.01	0.96	1.00	0.02	0.99	0.32		
YSCS	0.30	0.20	0.17	0.30	0.88	0.94	0.00	0.92	0.22	0.94	
YSCC	0.81	-0.09	-0.03	0.93	0.27	0.34	-0.03	0.32	-0.08	0.34	0.60

Correlations are shown between the following traits: protein (PP), fat (FP), lactose (LP) and useful yield (UP) content, total milk (YCUM), protein (PYCUM), fat (FYCUM), and lactose (LYCUM) yield until day 100, total somatic cell count (YSCC) and total cell score (YSCS) until day 100 (significance thresholds *r *> 0.159, *P *< 0.05; *r *> 0.208, *P *< 0.01)

Results for the two-QTL model conducted under QTL Express are presented in Table 4. Significant evidence for an additional QTL under a two-QTL model was found in three cases on OAR9 for UP, on OAR17 for SSC and on OAR26 for PP, with a difference of 112, 40, and 56 cM between the two loci, respectively. The two loci on OAR9 (UP) and OAR17 (SCC) were in coupling phase, whereas the QTL on OAR26 (PP) were of equal size and in repulsion phase. In an additional seven cases, the analyses by QTL Express suggested a significant second QTL located within 4 cM of the first QTL, and in six of these cases (the exception being the QTL for LYCUM on OAR22) the QTL were in repulsion phase of opposite and almost equal effect on OAR5 (SCS), OAR16 (YSCC) and OAR22 (FYCUM, LYCUM, PYCUM, YSCS). Furthermore, in none of these six cases, were QTL identified using the single QTL model. We suggest that the QTL identified using the two-QTL model in these cases maybe an artefact as the distance between the two loci is within one marker interval, and the QTL are of equal but opposite effect. Only the region identified using the two-QTL model for YCUM on OAR22 was in accordance with the locus identified using the single QTL model. In only three cases where a putative two-QTL model provided a better fit to the data than a single QTL model (UP on OAR9, SSC on OAR17, and PP on OAR26) were the intervals between QTL greater than the marker intervals, these chromosomes can therefore be considered as carrying two different linkage regions for the same trait (Table 4).

**Table 4:** Summary of significant QTL for lactation traits using QTL Express under a two QTL model

OAR	Trait	Position [cM]		QTL effect ± SE		Var^1 ^[%]	*F*-value		**Sign**^2^	
		
		QTL A	QTL B	QTL A	QTL B		2 vs0	2 vs 1	2vs0	2vs1
9	UP	12	124	-0.5 ± 0.2	-0.4 ± 0.1	7.8	7	7	*	*
17	SCC	32	72	216 ± 71	346 ± 87	8.4	8	8.4	**	**
26	PP	4	60	-0.1 ± 0.1	0.1 ± 0.1	7.9	7.2	7.8	*	*

Shown are the peak position of QTL A and QTL B for useful yield (UP) and protein (PP) content and somatic cell count (SCC); the QTL effect and the standard error (SE) of both QTL positions QTL A and QTL B (positions of each QTL are described by the average position in cM and further by the confidence interval from the bootstrapping analysis); ^1^variance of the phenotype explained by the QTL, ^2^significant threshold of the *F*-value (sign threshold) determines if the QTL reached the significance level under 2 vs 0 QTL (2 degrees of freedom), or 2 vs 1 QTL (1 degree of freedom); with *chromosome-wide *P *< 0.05; **chromosome-wide *P *< 0.01

From the interval mapping analyses, the observed QTL were detected with an average realised power 0.64 for *P *< 0.05 and. 0.4 for *P *< 0.01, using the method as described by Hu and Xu [37]. The greatest power was identified for the QTL for LYCUM, PYCUM and FYCUM on OAR3 (0.64 - 0.87 for *P *< 0.01 and 0.83 - 0.96 for *P *< 0.05). The lowest power of the detected QTL was found for the traits related to somatic cell count on OAR5, 11, 17, 22 and 23 (0.17 to 0.28 for *P *< 0.01 and 0.37 to 0.51 for *P *< 0.05).

### Meta-assembly

**Figure 5:**
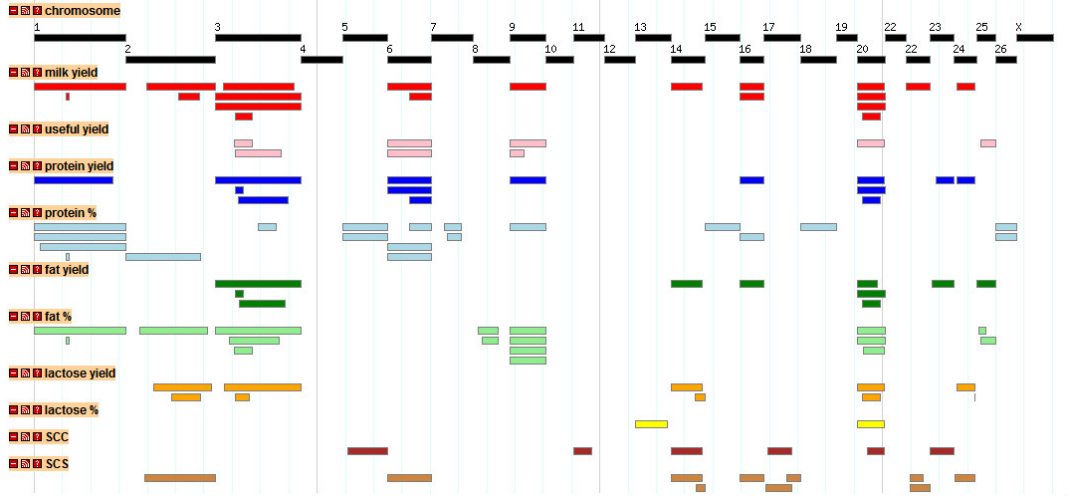
**Meta-assembly of ovine QTL**. Tracks available in the ReproGen ovine gbrowse website http://crcidp.vetsci.usyd.edu.au/cgi-bin/gbrowse/oaries_genome/ showing the sheep genomic locations of QTL for milk yield, useful yield protein yield and content, fat yield and content, lactose yield and content, and somatic cell score/count for individual QTL from this study and previous publications

In order to compare the QTL detected in this experiment with QTL findings in the public domain, a meta-assembly was conducted for each trait as shown in Figure 5. Among all studies, QTL for milk yield were identified on OAR1, 2, 3, 6, 9, 14, 16, 20, 22, and 24. Eight chromosomes were identified harbouring QTL for protein yield and six for fat yield. Eleven chromosomes were identified harbouring QTL for protein percent, of which five (OAR1, 5, 6, 7, and 26) were detected by at least two independent studies. QTL for fat percent were reported on seven chromosomes of which six (OAR1, 3, 8, 9, 20, and 25) were supported by at least two different studies. Limited QTL information has been reported for SCC and SCS. For lactose percent and useful yield this experiment represents the only published QTL. A detailed description of the QTL including peak QTL position and confidence interval (where available) from each study are shown in the public domain http://crcidp.vetsci.usyd.edu.au/cgi-bin/gbrowse/oaries_genome/. The information from all public domain QTL was not sufficiently dense to undertake a formal meta-analysis as conducted by Khatkar et al [13]. The meta-assembly provides a starting point for future meta-analysis as additional QTL studies will become available.

**Figure 6:**
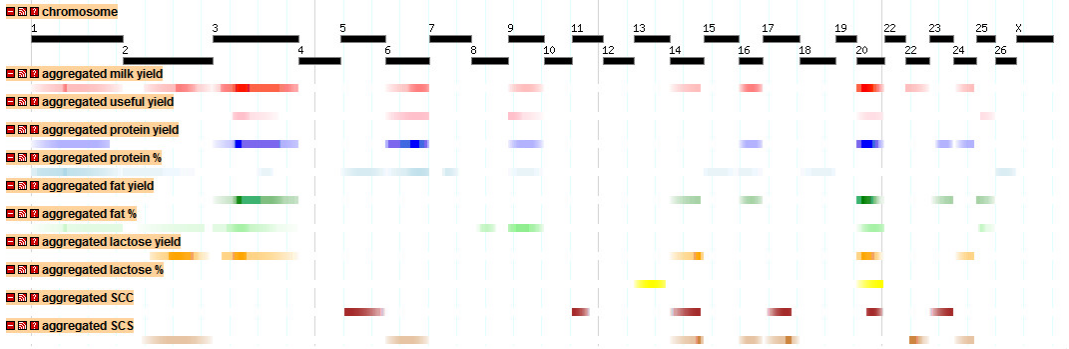
**Meta-assembly of ovine QTL**. Tracks available in the ReproGen ovine gbrowse website http://crcidp.vetsci.usyd.edu.au/cgi-bin/gbrowse/oaries_genome/ showing the sheep genomic locations of aggregate QTL for the same traits as in Fig. 5; each aggregate QTL is shown as a heatmap in which meta-score is proportional to colour intensity

The assembly of all milk QTL from the public domain is made available through an online browser where specific traits and all known QTL locations, are user selected. The browser allows within-trait and across-trait analysis of the information. An example for the main milk traits,, fat protein and lactose yield, and milk composition traits, protein, fat and lactose percentages, is shown in Figure 5. In order to aggregate the QTL information, the integrated QTL scores for each trait are shown in Figure 6 by way of a single QTL score with decreasing likelihood of QTL information away from peak location, and expressed as a heat map.

**Figure 7:**
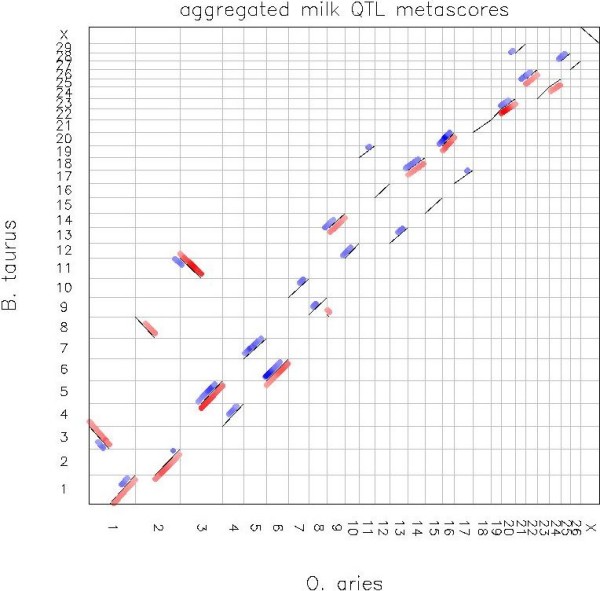
**Oxford Grid of ovine and bovine milk yield QTL meta-scores**. Oxford Grid comparison of ovine and bovine genomes; syntenic regions are shown as thin diagonal lines; aggregated QTL meta-scores for milk yield are also shown with meta-score proportional to colour intensity: sheep (red, to right of syntenic diagonals) and cow (blue, to left of diagonals)

In order to expand the information on significant QTL for lactation performance, the wealth of information derived from cattle studies was deemed to be suitable for a comparative genomic analysis. The results from all cattle QTL on lactation performance were compared by Oxford grid analyses to all known ovine lactation QTL as shown for the example of milk yield in Figure 7. Each grid permits a high resolution comparison on QTL identity, literature source and comparative position on either the bovine or ovine genome. From the comparative analysis, synteny was detected for QTL for milk yield across five chromosomes (BTA5-OAR3, BTA6-OAR6, BTA20-OAR16, BTA23-OAR20, and BTA26-OAR22). Similarly for protein and fat percent, seven syntenic regions were detected (BTA1-OAR1, BTA3-OAR1, BTA6-OAR6, BTA10-OAR7, BTA18-OAR14 for PP; and BTA14-OAR9, BTA28-OAR25 for FP). QTL in five comparative regions were also identified for somatic cell count/somatic cell score (BTA1-OAR1, BTA7-OAR5, BTA18-OAR14, BTA23-OAR20 and BTA26-OAR22).

## Discussion

This paper describes the lactation characteristics in a flock specifically designed for QTL mapping and reports on a number of important findings. Firstly it presents an appropriate model to describe lactation curve characteristics in sheep, secondly it confirms previously identified QTL on a population specific framework-map and identifies novel QTL for lactation, and finally it compares the location of all known QTL for lactation performance within sheep breeds and across sheep and cattle.

### Analysis of lactation data

The Wood model was shown to be appropriate to describe lactation curves which supports its earlier use in sheep [20,42-44]. However this is the first study in which the derived parameters were used in a QTL analysis for sheep. The use of quadratic lactation curve models provided a better fit to the lactation curves in dairy sheep when the evaluation of the lactation data was started after lactation day 30, but failed to give reasonable predictions of total milk yield [23]. Many different factors could be included in the nonlinear mixed model to help predict milk production. Each fixed effect used here was a significant factor in determining the lactation curve of each sheep. Previous studies have shown that various factors such as nutrition, multiple births, parity and lambing season have significant effects on milk production and hence affect model parameters [20,21,44,45]. The parameter estimates (*k*_*i*_, *b*_*i *_and *c*_*i*_) obtained in this study (5.35, 0.13 and 0.0032, respectively) cannot be directly compared with many other estimates from previous studies due to differences in measurement units. However, a study of lactation curves in dairy cows used the Wood [18] model to determine values for *k*_*i*_, *b*_*i *_and *c*_*i *_using the same units as those used here [46]. The estimates of the parameters from their research were 3.01 (*k*_*i*_), 0.091 (*b*_*i*_) and 0.0027 (*c*_*i*_) suggesting a general similarity between the two species in lactation curve characteristics. The most sizeable difference between both species is the first part of the lactation curve, which shows an early almost instantaneous peak of lactation in sheep, but a delayed peak in dairy cattle. In fact the coefficients *b*_*i *_and *c*_*i *_were found to be highly correlated (*r *= 0.79) in sheep, suggesting that one of the parameters may be redundant in this study.

The lactation phenotypes (protein, fat and lactose content and milk yield) derived from the parameters of the Wood model were similar to lactation data described by Gutierrez-Gil et al. and Theodorou et al [12,47]. We found that the SCS was similar to that reported previously [11]. This shows that the application of the Wood model is a useful tool to describe the overall lactation performance.

### QTL analysis and meta-assembly

This study is the fifth whole genome-scan which reports on QTL for lactation performance in sheep. This study is unique in that the experimental population used two breeds with extreme lactation characteristics. Previous studies were conducted across a diverse panel of breeds including Sarda × Lacaune, Lacaune, Churra and Basco-Bearnaise, Lacaune and Manech families [8,11,12,48,49]. Furthermore this study has anchored the QTL on a population-specific framework map therefore minimising ambiguity in the location of QTL, and used two approaches for QTL analyses, a maximum likelihood approach [27,33] and the regression method [35]. The QTL meta-assembly is a useful tool to show the greater QTL map for lactation traits in sheep among diverse experiments. The data suggest the existence of some major regions for milk production (yield, fat, protein, lactose). It is highly likely that the component traits are all influenced by QTL effects of milk yield which is further evidenced by the strong positive correlations between them (Table 3).

For the milk components expressed as percentage traits, we report for the first time on useful yield % as defined by Barillet [2]. Since this trait is a weighted index of fat and protein percentage it describes the overall milk characteristics for cheese making. The QTL for this trait was also aligned to QTL for milk yield. No QTL were observed for lactose percent which may be consistent with the very low degree of phenotypic variation observed in our study which was in accordance with some but not all other studies [29,47,50].

Although exact locations for QTL could not be compared given the generally very wide confidence intervals for such locations, the meta-assembly is useful in that a consensus location can be derived by combining QTL peak location and confidence intervals in a single score for multiple studies. This gives an aggregate profile of the most likely QTL location for traits with similar phenotype and background as shown in Figure 6. The meta-assembly can subsequently be used for a formal meta-analysis as undertaken by Khatkar et al. [13], but in the absence of such an analysis the QTL score seems appropriate. From our analyses, the statistical method used to identify QTL showed a high degree of consistency for the major (significant QTL) so comparisons across independent studies should not be adversely affected by using different analytical methods. However, reporting on QTL with a marginal significance may be affected by analysis methods, and the use of a least squares regression method perhaps over representing putative QTL. Despite the total number of reports on QTL analyses for lactation traits in sheep, most are derived from four populations and publications on fragments of the data (partial genome scans) do not add new information for meta-analyses over the genome-wide analyses [8,11,12,49,51]. Despite the breed diversity used in QTL mapping of lactation traits the consensus locations of major QTL for milk traits is remarkably consistent across breeds.

Comparison with the previous published results of a bovine meta-analysis revealed that almost all the linkage regions except the QTL for milk yield on OAR2 (BTA2) and for fat content on OAR8 (BTA9) are present both in sheep and cattle. We foresee the possibility of future studies to conduct a meta-analysis using data of both species. This will also enable the comparison of QTL for other milking traits such as lactation persistency where less information is available in either or both species. For genes relevant to lactation, Lemay et al. [52] examined gene loss and duplication, phylogeny, sequence conservation, and evolution using the genomes of a monotreme (platypus), a marsupial (opossum), and five placental mammals (bovine, human, dog, mice, rat). Compared with other genes in the bovine genome, milk and mammary genes are more likely to be present in all mammals; more likely to be duplicated in therians; more highly conserved across Mammalia; and evolving more slowly along the bovine lineage [52]. Their findings support the essentiality of milk to the survival of mammalian neonates and the establishment of milk secretory mechanisms more than 160 million years ago. Lactation has been studied more extensively in *Bos taurus *than in any other livestock species, resulting in extensive milk proteome data, identification of milk production QTL, and over 100,000 mammary-related bovine expressed sequence tags (ESTs). The availability of the bovine genome sequence provides unique opportunities to investigate milk and lactation traits. With a known gene density for regions underlying the broad confidence intervals of QTL associated with lactation, each individual QTL is expected to contain between 105 and 127 positional candidate genes [52]. However, cross-species synteny in QTL location and general conservation of lactation genes across Mammalia, suggests that common ancestral genes may be responsible for variation in lactation performance. Both copy number and sequence variation are likely to contribute to the diversity of milk protein composition across species [52]. Therefore, further comparisons of sheep and cattle QTL through the use of high density SNP analysis in both species may identify common ancestral genes and chromosomal segments.

Despite high resolution fine-mapping tools, the identification of causal genes that underlie QTL remains a challenging task. The use of functional information on lactation networks may in part offer additional insight in selecting positional-functional candidate genes in fine-mapped regions. The use of improved QTL software system such as QTL MatchMaker to integrate and mine QTL information across human, mouse and rat genomes and to annotate functional genomic data [53] should accelerate the discovery of causative genes underlying QTL. This will also in part elucidate the question if genes are physically clustered with respect to their known functions or phenotypic effects [54].

## Conclusion

In conclusion, we showed that the application of a mathematical model describing lactation curves of individual ewes is a useful tool to describe milk production of sheep which can then be used for QTL mapping. There is a high degree of consistency of QTL across breeds particular on OAR3, 6, 20 among diverse studies. The analysis of such studies can the streamlined by online meta-assemblies supported by appropriate browsers. We also present evidence for a number of novel QTL for various milk production traits, from which most (except QTL for milk yield on OAR2 and for fat content on OAR8) were in agreement with QTL mapped to corresponding bovine genome regions. We show the possibility of future studies to conduct a meta-analysis using data of both species. Our data supports evidence that there are ancestral alleles across ruminants which will be eventually confirmed by cross-species meta-assemblies as reported here.

## Competing interests

The authors declare that they have no competing interests.

## Authors' contributions

HWR was responsible for the overall design, project management, and was involved in analysis of the data and writing the manuscript. EJ ran the final models and QTL analyses, participated the manuscript preparation. DMcG carried out the analysis of the lactation curves, developed the models and helped drafting the manuscript. MH calculated the QTL score and designed the online data source and prepared data for the oxford grid. MKL ran the early stage QTL analyses, was responsible for the data assembly, and the phenotypic analysis. PCT developed the statistical methodology for the lactation curve analysis and QTL methodology, implemented the QTL-MLE program, and contributed to manuscript preparation and the overall design. All authors read and approved the final manuscript.

## Supplementary Material

Additional file 1 **Correlation between results of the Wood model applied to lactations of different length**. The Wood model was fitted to observed lactation data, truncated at days 50, 80, 100, 150, 200, 250 and 300. The correlation between the cumulative yield using lactations of 100 days length (*x*-axis) is plotted against predicted cumulative yield of lactation length shorter (50 and 80) and longer (150, 200, 250, 300, and 400 days) than 100 days (*y*-axis). Click here for file

Additional file 2 **Summary of QTL for lactation traits using QTL Express single QTL models**. Shown are the QTL relative QTL position and the confidence interval (CI) along the ^1^male distance map [27] for the following traits: protein (PP), fat (FP), lactose (LP) and useful yield (UP) content, total milk (YCUM), protein (PYCUM), fat (FYCUM), and lactose (LYCUM) yield until day 100, total somatic cell count (YSCC) and total cell score (YSCS) until day 100; the F-value and; ^2^significant threshold of the *F*-value (Sign.) which determines if the QTL reached the significance level with *chromosome-wide *P *< 0.05; **chromosome-wide *P *< 0.01; ***experiment-wide *P *< 0.05; ****experiment-wide *P *< 0.01; ^3^QTL heritability: proportion of the phenotypic variance accounted for by the QTL [1-(MS of full model/MS of reduced model)]. Click here for file
