# Supplementary material for: Mapping quantitative trait loci (QTL) in sheep. II. Meta-assembly and identification of novel QTL for milk production traits in sheep
Source: Genet Sel Evol. 2009 Oct 22;41(1):45. doi: 10.1186/1297-9686-41-45 (PMC2772855; doi:10.1186/1297-9686-41-45)

Additional file 1

File format: PDF

Title of data: Correlation between results of the Wood model applied to lactations of different length

Description: The Wood model was fitted to observed lactation data, truncated at days 50, 80, 100, 150, 200, 250 and 300. The correlation between the cumulative yield using lactations of 100 days length (*x*-axis) is plotted against predicted cumulative yield of lactation length shorter (50 and 80) and longer (150, 200, 250, 300, and 400 days) than 100 days (*y*-axis).

Trait [data until day]	YCUM(100) [total lactation]
YCUM(50) [total lactation]	0.99
YCUM(50) [day50]	0.99
YCUM(50) [day80]	0.99
YCUM(50) [day100]	0.99
YCUM(50) [day150]	0.99
YCUM(50) [day200]	0.99
YCUM(50) [day250]	0.99
YCUM(50) [day300]	0.99
YCUM(50) [day400]	0.99

Trait	YCUM(100) [total lactation]
YCUM(80) [total lactation]	1.00
YCUM(80) [day50]	0.99
YCUM(80) [day80]	1.00
YCUM(80) [day100]	1.00
YCUM(80) [day150]	1.00
YCUM(80) [day200]	1.00
YCUM(80) [day250]	1.00
YCUM(80) [day300]	1.00
YCUM(80) [day400]	1.00

Trait	YCUM(100) [total lactation]
YCUM(100) [total lactation]	1.00
YCUM(100) [day50]	0.98
YCUM(100) [day80]	1.00
YCUM(100) [day100]	1.00
YCUM(100) [day150]	1.00
YCUM(100) [day200]	1.00
YCUM(100) [day250]	1.00
YCUM(100) [day300]	1.00
YCUM(100) [day400]	1.00

Trait	YCUM(100) [total lactation]
YCUM(150) [total lactation]	1.00
YCUM(150) [day50]	0.95
YCUM(150) [day80]	0.98
YCUM(150) [day100]	0.99
YCUM(150) [day150]	0.99
YCUM(150) [day200]	1.00
YCUM(150) [day250]	1.00
YCUM(150) [day300]	0.99
YCUM(150) [day400]	0.99

Trait	YCUM(100) [total lactation]
YCUM(200) [total lactation]	0.98
YCUM(200) [day50]	0.91
YCUM(200) [day80]	0.96
YCUM(200) [day100]	0.97
YCUM(200) [day150]	0.98
YCUM(200) [day200]	0.98
YCUM(200) [day250]	0.98
YCUM(200) [day300]	0.98
YCUM(200) [day400]	0.98

Trait	YCUM(100) [total lactation]
YCUM(250) [total lactation]	0.96
YCUM(250) [day50]	0.87
YCUM(250) [day80]	0.92
YCUM(250) [day100]	0.95
YCUM(250) [day150]	0.96
YCUM(250) [day200]	0.96
YCUM(250) [day250]	0.96
YCUM(250) [day300]	0.96
YCUM(250) [day400]	0.96

Trait	YCUM(100) [total lactation]
YCUM(300) [total lactation]	0.94
YCUM(300) [day50]	0.83
YCUM(300) [day80]	0.88
YCUM(300) [day100]	0.92
YCUM(300) [day150]	0.94
YCUM(300) [day200]	0.94
YCUM(300) [day250]	0.94
YCUM(300) [day300]	0.94
YCUM(300) [day400]	0.94

Trait	YCUM(100) [total lactation]
YCUM(400) [total lactation]	0.90
YCUM(400) [day50]	0.76
YCUM(400) [day80]	0.81
YCUM(400) [day100]	0.85
YCUM(400) [day150]	0.89
YCUM(400) [day200]	0.90
YCUM(400) [day250]	0.90
YCUM(400) [day300]	0.90
YCUM(400) [day400]	0.90

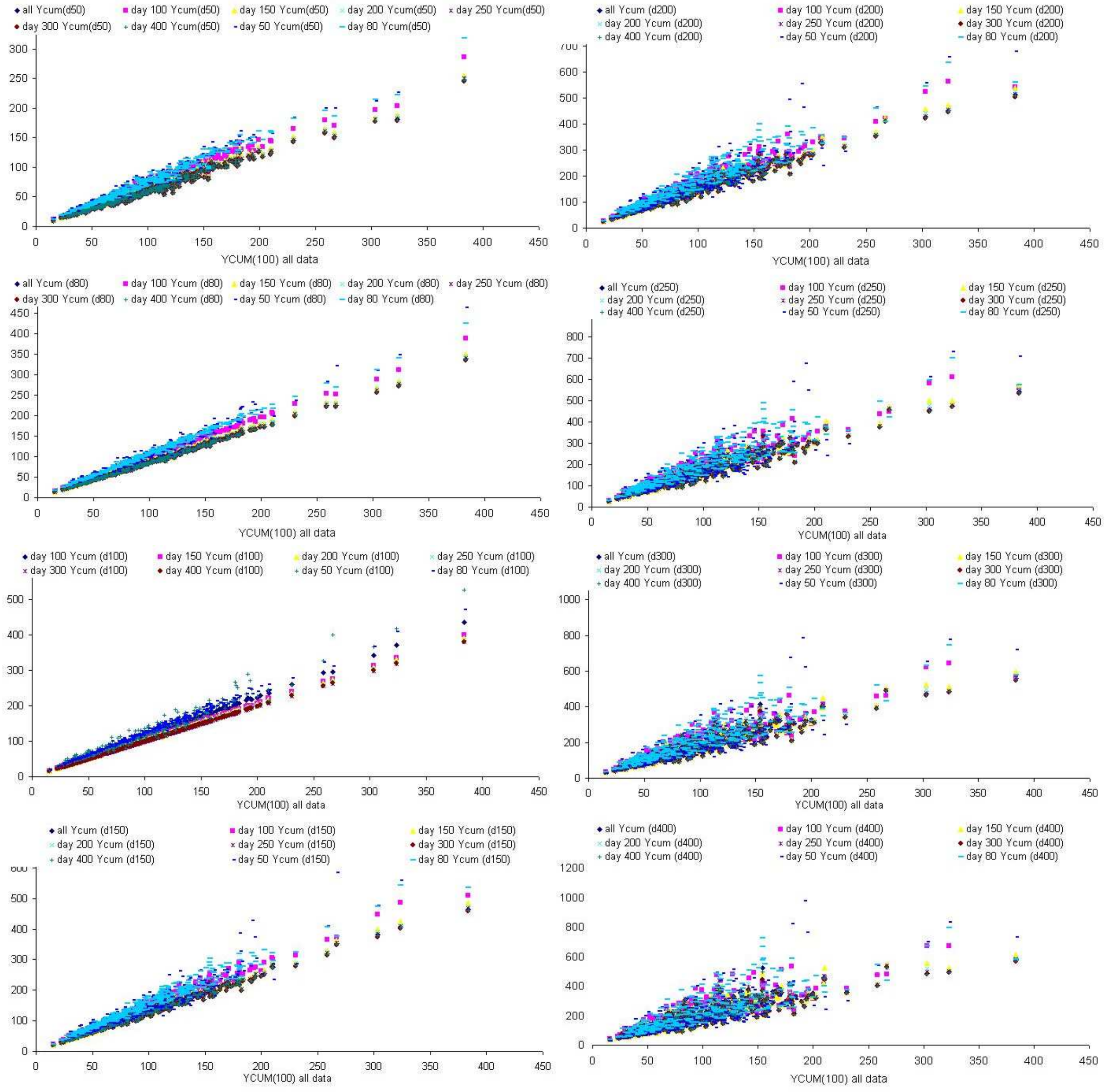

Supplement: Additional file 1 — Correlation between results of the Wood model applied to lactations of different length. The Wood model was fitted to observed lactation data, truncated at days 50, 80, 100, 150, 200, 250 and 300. The correlation between the cumulative yield using lactations of 100 days length (x-axis) is plotted against predicted cumulative yield of lactation length shorter (50 and 80) and longer (150, 200, 250, 300, and 400 days) than 100 days (y-axis). [file 1297-9686-41-45-S1.PDF]
