# Supplementary material for: Mapping quantitative trait loci (QTL) in sheep. II. Meta-assembly and identification of novel QTL for milk production traits in sheep
Source: Genet Sel Evol. 2009 Oct 22;41(1):45. doi: 10.1186/1297-9686-41-45 (PMC2772855; doi:10.1186/1297-9686-41-45)
Supplement: Additional file 2 — Summary of QTL for lactation traits using QTL Express single QTL models. Shown are the QTL relative QTL position and the confidence interval (CI) along the 1male distance map [27] for the following traits: protein (PP), fat (FP), lactose (LP) and useful yield (UP) content, total milk (YCUM), protein (PYCUM), fat (FYCUM), and lactose (LYCUM) yield until day 100, total somatic cell count (YSCC) and total cell score (YSCS) until day 100; the F-value and; 2significant threshold of the F-value (Sign.) which determines if the QTL reached the significance level with *chromosome-wide P < 0.05; **chromosome-wide P < 0.01; ***experiment-wide P < 0.05; ****experiment-wide P < 0.01; 3QTL heritability: proportion of the phenotypic variance accounted for by the QTL [1-(MS of full model/MS of reduced model)]. [file 1297-9686-41-45-S2.PDF]

Additional file 2

File format: PDF

Title of data: Summary of QTL for lactation traits using QTL Express single QTL models.

Description: Shown are the QTL relative QTL position and the confidence interval (CI) along the [1]male distance map [27] for the following traits: protein (PP), fat (FP), lactose (LP) and useful yield (UP) content, total milk (YCUM), protein (PYCUM), fat (FYCUM), and lactose (LYCUM) yield until day 100, total somatic cell count (YSCC) and total cell score (YSCS) until day 100; the F-value and; [2]significant threshold of the *F*-value (Sign.) which determines if the QTL reached the significance level with *chromosome-wide $P < 0.05$; **chromosome-wide $P < 0.01$; ***experiment-wide $P < 0.05$; ****experiment-wide $P < 0.01$; [3]QTL heritability: proportion of the phenotypic variance accounted for by the QTL [1-(MS of full model/ MS of reduced model)]

OAR	Trait	QTL position [cM]		CI [cM]		*F*-value	Sign[2]	QTL[3] [%]
		Peak[1]	marker	upper[1]	lower[1]			
1	PP	133	RM065	30	348	8.7	*	5.1
2	LYCUM	215	CSRD254	98	297	9.8	*	5.8
2	YCUM	217	CSRD254	73	300	9.7	*	5.4
2	YSCS	217	CSRD254	66	300	8.4	*	4.8
3	FP	95	DIK4796	49	243	10.6	*	5.9
3	FYCUM	96	DIK4796	79	238	17.9	****	10.7
3	LYCUM	96	DIK4796	28	289	11.4	*	6.9
3	PYCUM	94	DIK4796	74	285	13	**	8.1
3	UP	96	DIK4796	80	250	8.4	*	4.9
3	YCUM	96	DIK4796	38	302	9.6	*	5.4
5	SCC	27	MCM380	9.5	164	5.1	*	2.6
6	PP	126	BM4311	0	158	7.2	*	4.1
6	UP	125	BM4311	0	149	7.7	*	4.4
7	PP	77	MCM223	48	122	11.1	**	6.6
8	FP	38	UWCA9	13	93	7.4	*	4.0

9	UP	11	ETH225	0	132	6.9	*	3.9
11	SCC	0	HEL10	0	63	5.1	*	2.6
13	LP	63	HUJ616	0	114	2.4	*	NA
14	FYCUM	100	MCMA19	11	100	7.6	*	4.4
14	LYCUM	100	MCMA19	7	100	7.8	*	4.6
14	YCUM	100	MCMA19	10	100	6.9	*	3.7
14	YSCS	100	MCMA19	6	100	9.2	*	5.3
14	YSCC	96	MCMA19	0	100	6.1	*	3.4
17	SCS	81	TGLA322	4	96	10.1	**	5.5
17	SCC	82	TGLA322	15	96	7.3	*	3.9
20	FYCUM	17	INRA132	0	62	13.1	**	7.8
20	LP	108	MCMA23	0	112	2.4	*	NA
20	LYCUM	15	INRA132	0	112	10.5	**	6.3
20	PYCUM	13	INRA132	0	112	11.4	**	7.1
20	UP	62	CSRD226	0	87	7.2	*	4.2
20	YCUM	19	INRA132	0	111	9.	*	5
22	LYCUM	32	BMS907	15	84	7.2	*	4.2
22	YCUM	83	MAF92	4.5	84	6.5	*	3.5
22	YSCS	39	BMS907	19	83	9.9	*	5.8
23	YSCC	80	URB0031	0	80	6.1	*	3.5
24	LYCUM	99	DIK5147	8	99	8.5	*	5
24	PYCUM	99	DIK5147	11	99	7.3	*	4.4
24	YCUM	99	DIK5147	5.5	99	8.9	*	5
24	YSCS	99	DIK5147	5	99	6.6	*	3.7
25	FP	21	DIK2451	15	74	7.6	*	4.2
25	UP	21	DIK2451	13	69	8.4	*	4.9
26	PP	0	BMS2168	0	62	6.2	*	3.5
